# The effect of combined drought and trace metal elements stress on the physiological response of three *Miscanthus* hybrids

**DOI:** 10.1038/s41598-023-37564-5

**Published:** 2023-06-28

**Authors:** Jacek Krzyżak, Szymon Rusinowski, Krzysztof Sitko, Alicja Szada-Borzyszkowska, Radosław Stec, Paulina Janota, Elaine Jensen, Andreas Kiesel, Marta Pogrzeba

**Affiliations:** 1 Institute for Ecology of Industrial Areas, 6 Kossutha Street, 40-844 Katowice, Poland; 2 Plant Ecophysiology Team, University of Silesia in Katowice, 28 Jagiellońska Street, 40-032 Katowice, Poland; 3 Institute of Biological, Environmental and Rural Sciences, Aberystwyth University, Plas Gogerddan, Aberystwyth, SY23 3EB UK; 4 Biobased Resources in the Bioeconomy (340B), Institute of Crop Science, University of Hohenheim, Stuttgart, Germany

**Keywords:** Plant sciences, Biofuels, Photosynthesis, Plant ecology, Plant physiology, Plant stress responses

## Abstract

Drought is a serious threat worldwide and has a significant impact on agricultural production and soil health. It can pose an even greater threat when it involves land contaminated with trace metal element (TMEs). To prevent desertification, such land should be properly managed and growing *Miscanthus* for energy or raw material purposes could be a solution. The effects of drought and TMEs were studied in a pot experiment on three different *Miscanthus* hybrids (conventional *Miscanthus* × *giganteus*, TV1 and GNT10) considering growth parameters, photosynthetic parameters and elemental composition of roots, rhizomes and shoots. GNT10 was characterised by the weakest gas exchange among the hybrids, which was compensated by the highest number of leaves and biomass. The strongest correlations between the studied parameters were found for TV1, which might indicate a high sensitivity to TME stress. For M × g and GNT10, the main mechanisms for coping with stress seem to be biomass management through number of shoots and leaves and gas exchange. The main factor determining the extent of accumulation of TMEs was the amount of water applied in the experimental treatment, which was related to the location of the plant in the aniso-isohydric continuum. GNT10 was the most resistant plant to combined stress, while it responded similarly to TV1 when drought and trace metal elements were applied separately.

## Introduction

Drought is a serious threat worldwide, especially in climates with extremely high temperatures^1,2^. Apart from its impact on drinking water supply and quality in some regions^3^, drought has significant implications for agricultural production and soil health^4,5^. In areas with additional environmental problems such as nutrient deficiencies, high sand or stone content, or contamination, drought can have even more negative impacts. Vicente-Serrano et al.^1^ emphasise the importance of human influence on the occurrence of droughts in the environment, especially through human interaction with the environment, i.e. misuse of environmental resources. This influence is particularly important when agricultural land is classified as marginal. According to Bury et al.^6^, who reviewed the various definitions of marginal land, the definition proposed by USDA-NRCS^7^ may be the one most associated with environmentally induced drought. In this definition, marginal land is described as "unlike prime cropland, marginal land has a poor combination of soil physical and chemical properties for food, feed and fodder production". Frequent drought phenomena in such areas can lead to progressive soil erosion and consequent desertification of the land. Eventually, such land becomes worthless from an economic perspective^8,9^. For this reason, there is a tremendous need for proper care for drought-prone marginal lands. As long as agricultural production on such land is not profitable or impossible for other reasons, such as contamination, growing energy crops is a viable alternative^6,10,11^. Among the various energy crops that can be grown on such marginal soils, there are C4 grasses that successfully cope with drought and have low nutrient requirements^12,13^. The relationship between abiotic stress and plant growth and development is currently an important topic, especially due to rapid climate change. Published data mostly refer to a combination of heat and drought stress^14–16^. There is limited work on the effects of drought and trace metal elements (TME)^2,17,18^, especially for energy crop species^6,19,20^ that could be grown on TME-contaminated soils without risk to humans. Rusinowski et al.^21^ showed that the influence of TME-contaminated soils on the photosynthetic apparatus of three C4 grasses (*Miscanthus* × *giganteus*, *Panicum virgatum* and *Spartina pectinata*) was low when measured in field-cultivated mature plantations. However, environmental conditions associated with drought and heat during the growing season favoured biomass production of *M.* × *giganteus*. Despite the low accumulation and the undeniable potential application of *Miscanthus* in the phytostabilisation process of TME-contaminated soils, further field work was conducted with different seed-based *Miscanthus* hybrids^19^. Here, *M*. × *giganteus* showed different water use behaviour than the hybrids studied in field cultivation during periods of limited water availability, with TME (Cd, Zn) accumulation in shoots being higher in the less water-conservative *M*. × *giganteus*. Since field trials are limited in terms of experimental design and control of water deficit, there was a need to describe the observed mechanism using data from the trial conducted under controlled conditions. Thus, abiotic stresses (i.e. water deficit and TMEs) can be applied individually and in combination, while the effects on plant growth and development are studied. In the present study, we investigate the effects of drought and TMEs on three different *Miscanthus* hybrids on growth parameters, the activity and efficiency of the photosynthetic apparatus and the accumulation of elements in roots, rhizomes and shoots.

## Results

### Soil physico-chemical characteristics

Overall, the physico-chemical properties of the soil in the uncontaminated soil (C) were worse compared to the contaminated soil from Bytom (Table 1). The pH of C was 6.13, while it was about neutral in Bytom. OM was almost half that of Bytom, and several elements such as assimilable P, Mg, Ca, total P and total K were higher in the soil from Bytom. EC measurements also reflected the poorer fertility of the sandy control soil. The Bytom soil was heavily polluted with TMEs and contained 20, 32 and 36 times higher levels of Pb, Cd and Zn, respectively, compared to the control soil.

**Table 1:** Soil physicochemical parameters of Pińczów non-contaminated (C) and Bytom trace metal element contaminated (B) soils.

	C	B
Physicochemical parameters		
pH (H_2_O)	6.13 ± 0.01 b	7.2 ± 0.01 a
pH (KCl)	5.16 ± 0.01 b	6.47 ± 0.02 a
EC (µS cm^−1^)	55.8 ± 0.2 b	86.5 ± 4.8 a
OM (%)	3.32 ± 0.04 b	6.03 ± 0.10 a

Soil texture	Sandy	Silty loam
Element composition		
N (% DW)	0.24 ± 0.02 a	0.22 ± 0.00 a
P (mg P_2_O_5_ 100 g^−1^)	2.5 ± 0.1 b	5.4 ± 0.5 a
K (mg K_2_O 100 g^−1^)	16.2 ± 0.2 b	24.2 ± 0.6 a
Pb (mg kg^−1^)	20.2 ± 1.7 b	418.3 ± 7.6 a
Cd (mg kg^−1^)	0.5 ± 0.0 b	16.2 ± 0.2 a
Zn (mg kg^−1^)	52 ± 1 b	1862 ± 25 a
Fe (mg kg^−1^)	3590 ± 120 b	13,680 ± 40 a
Mg (mg kg^−1^)	640 ± 10 b	2420 ± 40 a
Ca (mg kg^−1^)	1380 ± 50 b	4040 ± 3 a
P (mg kg^−1^)	310 ± 10 b	769 ± 12 a
K (mg kg^−1^)	644 ± 12 b	1292 ± 46 a
S (%)	0.01 ± 0.00 b	0.03 ± 0.00 a

Presented values are mean ± SE (n = 5). Lower case letters denote significant differences between treatments according to Mann–Whitney U test (*p* > 0.05).

### Biomass production and other growth parameters

There were no significant differences between the tested *Miscanthus* hybrids in shoot height and diameter under drought and/or TME stress (Table 2). M × g was characterised by a significantly lower number and biomass of shoots on soil contaminated with TMEs in combination with drought compared to the control. Among the genotypes tested on TME-contaminated soil, the growth of TV1 was most affected. The number of shoots of TV1, water content, biomass and number of leaves per shoot were significantly lower on the contaminated soils compared to the control soils. For GNT10, the decrease in shoot biomass was measured on contaminated soil compared to the control (Table 2). It is worth noting that GNT10 had the highest shoot biomass on the well-watered control soil among the hybrids tested (Table 2).

**Table 2:** The effect of drought (/30) or/and trace metal element contamination (B/) on selected growth parameters of three tested *Miscanthus* hybrids (M × g, TV1, and GNT10).

	C/M/100	B/M/100	C/M/30	B/M/30	C/T/100	B/T/100	C/T/30	B/T/30	C/G/100	B/G/100	C/G/30	B/G/30
Height of shoot (cm)	71.0 ± 4.5 bc	75.7 ± 1.6 abc	69.0 ± 5.9 bc	57.0 ± 11.8 c	86.2 ± 4.0 abc	86.0 ± 8.9 abc	88.0 ± 18.6 abc	80.2 ± 6.1 ab	105.0 ± 8.4 a	72.5 ± 2.8 bc	94.3 ± 8.6 ab	87.3 ± 6.3 abc
Diameter of shoot (cm)	6.7 ± 0.3 ab	6.2 ± 0.4 ab	5.7 ± 0.5 ab	5.1 ± 0.9 b	5.8 ± 0.3 ab	6.5 ± 0.6 ab	5.2 ± 0.3 b	5.8 ± 0.4 ab	7.2 ± 0.5 a	6.2 ± 0.5 ab	7.3 ± 0.6 a	6.8 ± 0.4 ab
Number of shoots	2.8 ± 0.4 bc	1.7 ± 0.2 cd	2.7 ± 0.2 bcd	1.0 ± 0.5 d	6.0 ± 0.8 a	3.0 ± 0.0 bc	4.8 ± 0.4 a	2.5 ± 0.4 bcd	3.3 ± 0.2 b	2.3 ± 0.4 bcd	2.7 ± 0.2 bcd	2.0 ± 0.4 bcd
Number of leaves per shoot	11.1 ± 1.0 ab	10.7 ± 0.7 ab	9.5 ± 0.7 bc	9.0 ± 1.4 bcd	9.5 ± 0.1 bc	6.8 ± 0.3 d	7.4 ± 0.1 cd	7.1 ± 0.6 cd	12.5 ± 0.2 a	12.8 ± 0.6 a	12.4 ± 1.3 a	12.6 ± 1.0 a
Content of H_2_O in shoots (%)	75.9 ± 2.5 ab	73.3 ± 0.8 b ab	75.7 ± 2.3 ab	69.7 ± 4.6 bc	74.7 ± 1.1 ab	66.4 ± 3.4 c	71.2 ± 1.5 bc	70.9 ± 2.1 bc	78.4 ± 1.0 ab	81.0 ± 1.0 a	79.8 ± 1.9 a	74.4 ± 2.7ab
Biomass of shoots (g FW)	58.2 ± 7.9 cd	35.3 ± 6.2 ef	42.4 ± 2.5 de	12.0 ± 5.0 f.	79.9 ± 6.0 b	33.9 ± 2.5 ef	61.9 ± 3.2 bcd	28.5 ± 2.1 ef	101.8 ± 3.8 a	57.0 ± 12.0 cd	70.4 ± 11.0 bc	54.5 ± 1.8 cd
Biomass of shoots (g DW)	14.5 ± 2.9 bc	9.3 ± 1.6 cde	10.5 ± 0.8 cde	3.2 ± 2.8 e	20.4 ± 2.1 ab	11.2 ± 1.1 cde	17.8 ± 0.8 ab	8.2 ± 0.5 cd	21.9 ± 0.6 a	11.2 ± 2.7 cde	15.0 ± 3.7 ab	13.8 ± 1.3 bcd
Biomass of rhizomes (g DW)	8.0 ± 1.9 ab	6.9 ± 0.6 ab	6.8 ± 2.9 ab	8.2 ± 0.9 ab	11.7 ± 2.6 ab	10.2 ± 2.1 ab	13.0 ± 1.5 a	12.1 ± 2.0 ab	9.8 ± 0.9 ab	6.4 ± 0.7 b	8.0 ± 1.7 ab	11.1 ± 1.8 ab
Biomass of roots (g DW)	3.3 ± 1.0 b	3.3 ± 0.7 b	3.0 ± 0.8 b	1.8 ± 0.3 b	4.8 ± 1.1 b	4.4 ± 0.7 ab	7.1 ± 1.0 a	3.5 ± 0.7 b	3.0 ± 0.5 b	2.5 ± 0.1 b	2.4 ± 0.5 b	2.3 ± 0.5 b

C—control soil, B—contaminated soil, M (M × g) —*Miscanthus* × *giganteus*, T (TV1)—Athena Terravesta, Ltd., G (GNT10)—clonal interspecific hybrid, 30—drought conditions (30% of RWC), 100—well-watered control (100% RWC). Presented values are mean ± SE (n = 4). Lower case letters denote significant differences between treatments according to Fisher’s LSD test (p > 0.05).

### Gas exchange

**Figure 1:**
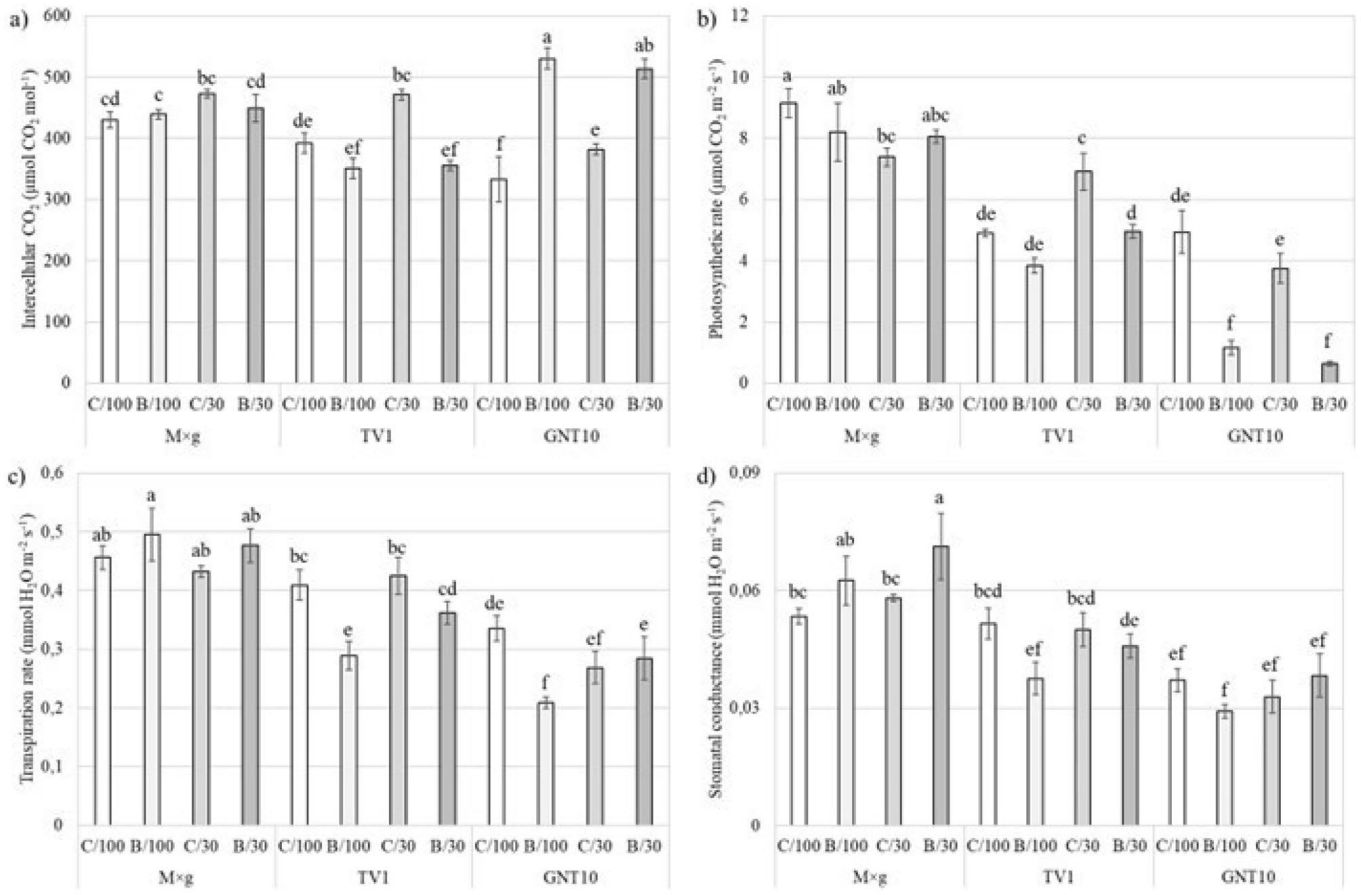
The effect of drought (/30) or/and TME contamination (B/) on photosynthetic rate (**a**), intercellular CO_2_ (**b**), transpiration rate (**c**), and stomatal conductance (**d**) of three tested *Miscanthus* hybrids (M × g, TV1, and GNT10). Presented values are mean ± SE (n = 20).

M × g had a significantly lower photosynthetic rate on the drought control soil (C/30), although transpiration and intercellular concentration did not change significantly between the experimental variants at the same time (Fig. 1). The combination of drought and TME caused a significant increase in stomatal conductance in M × g compared to the control soil. Drought caused a significant increase in photosynthetic and transpiration rate in TV1, while TME significantly decreased stomatal conductance and transpiration rate compared to the control. Accumulation of TMEs in GNT10 tissues significantly decreased the photosynthetic rate and increased the intercellular CO_2_ concentration compared to the control (Fig. 1).

### Pigment content

**Figure 2:**
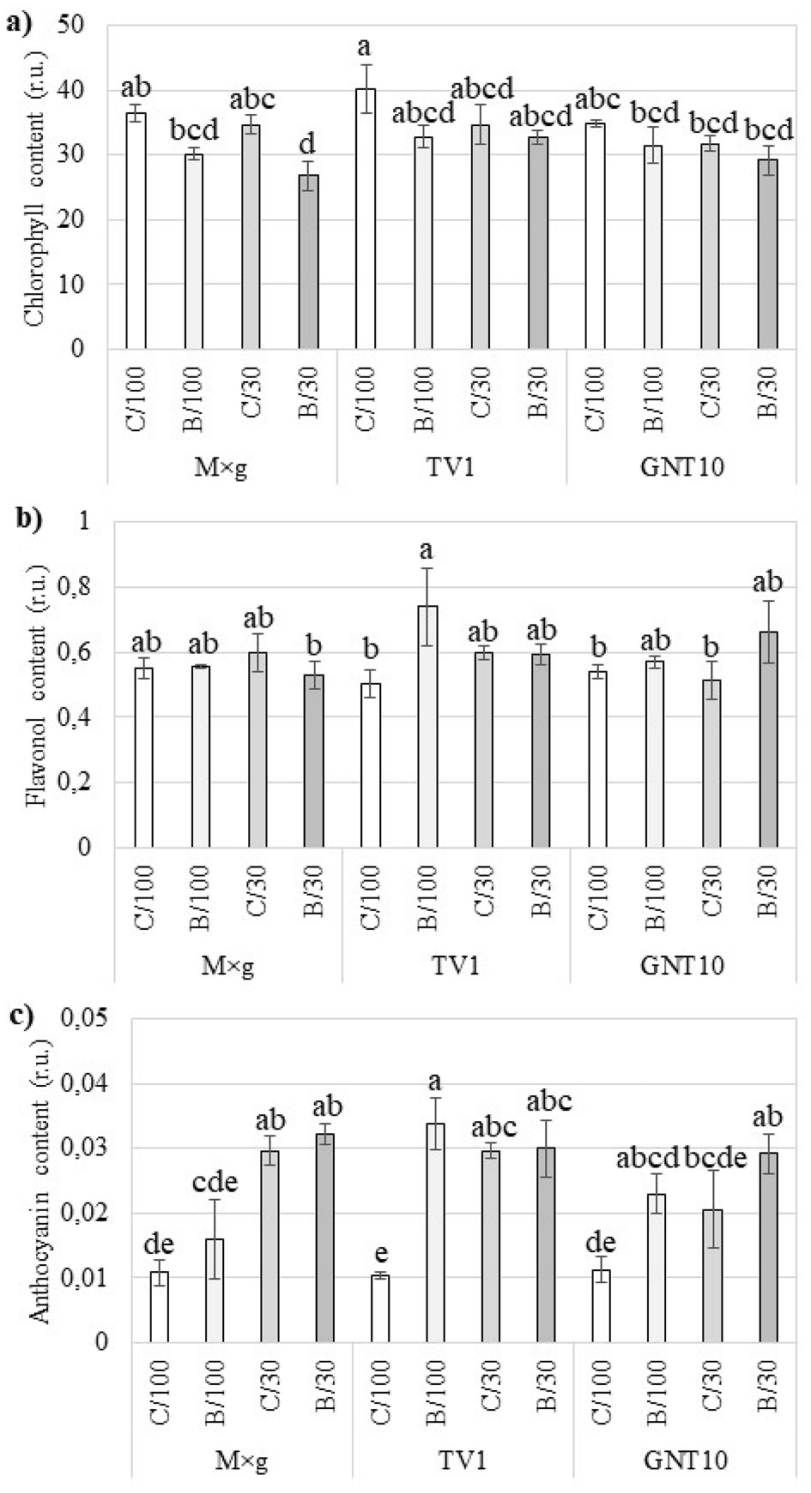
The effect of drought (/30) or/and TME contamination (B/) on chlorophyll (**a**), flavonol (**b**), and anthocyanin content (**c**) of three tested *Miscanthus* hybrids (M × g, TV1, and GNT10). Presented values are means ± SE (n = 5).

The significant decrease in chlorophyll content under stress conditions was observed only in M × g under the combined drought and TME treatment (Fig. 2A). The other hybrids tested did not show significant changes in chlorophyll content of leaves under stress. TV1 showed a significant increase in flavanol content in leaves in TME-contaminated, well-watered soil compared to the control (Fig. 2B). The other changes observed for each *Miscanthus* hybrid tested were not statistically significant. M × g was characterised by a significant increase in anthocyanin content in leaves under drought conditions compared to the control (Fig. 2C). In TV1, the anthocyanin content increased significantly in all experimental variants compared to the control. GNT10, on the other hand, only showed a significant increase in anthocyanin content in the leaves in the combined stress variant compared to the control (Fig. 2C).

### Element concentration

**Figure 3:**
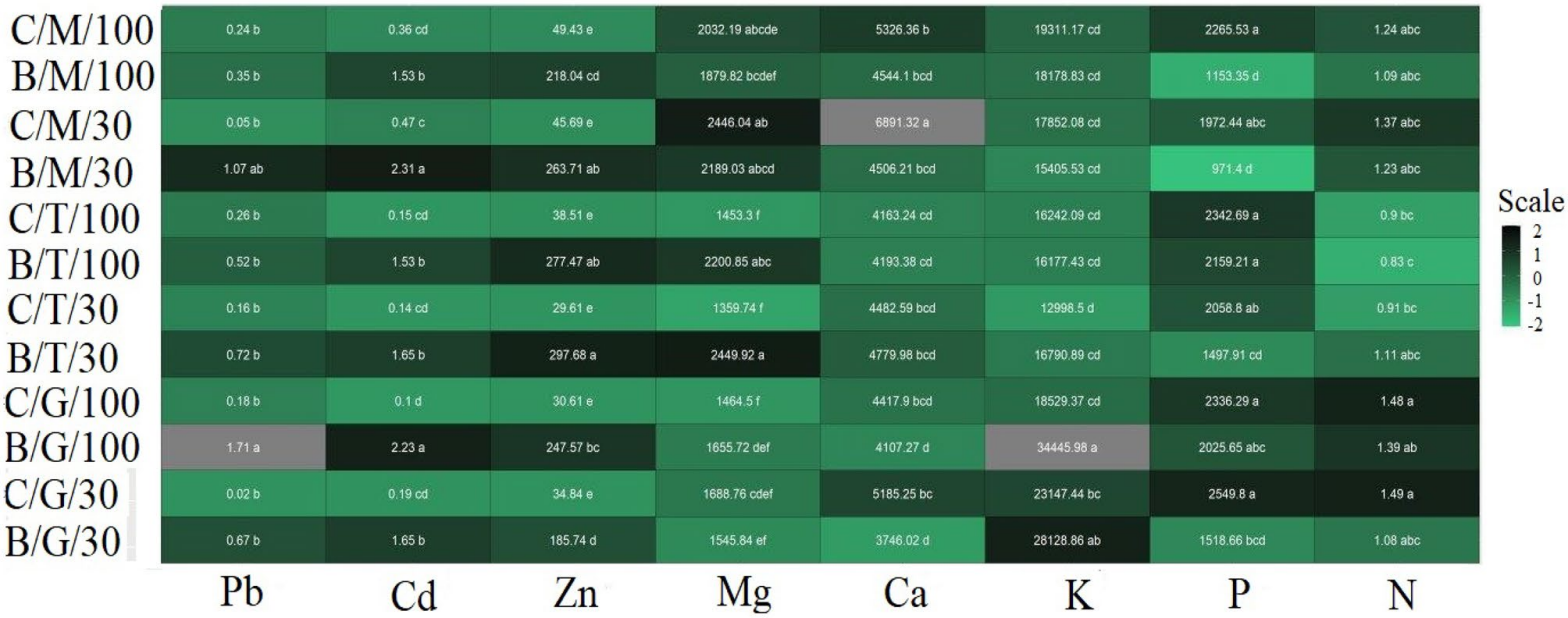
The effect of drought (/30) or/and TME contamination (B/), versus fully watered uncontaminated control soil (C/100) on accumulation of elements in shoots (µg g^−1^ DW) of three tested *Miscanthus* hybrids. Presented values are means ± SE (n = 4).

GNT10, which grew on well-watered soil, accumulated a significantly higher Pb concentration in the shoots than all other hybrids tested (Fig. 3). In the roots and rhizomes of all tested hybrids, the accumulation of lead on metal-containing soil was significantly higher than in the control (Figure S1 and S2).

The *Miscanthus* hybrids studied were characterised by a different strategy of accumulation of cadmium and zinc in the shoots on metalliferous soil in response to drought (Fig. 3). M × g accumulated significantly more Cd and Zn in shoots in the B/30 drought variant than in the B/100 variant, while GNT10 behaved inversely. In TV1, no significant differences in the accumulation of the two TMEs were measured between the B/30 and B/100 variants. Similar ratios as for the accumulation in the shoots were observed for the accumulation of Cd and Zn in the roots and rhizomes of the hybrids studied (Figure S1 and S2).

M × g showed the highest accumulation of calcium and magnesium in leaves on clean soil under drought conditions C/30 (Fig. 3). At the same time, the highest concentrations of calcium were accumulated in the roots and rhizomes of M × g in the B/30 variant (Figure S1 and S2). In the leaves of TV1, a significantly higher Mg concentration was found in the samples grown on the soil containing metals, irrespective of drought, while no differences were found in Ca accumulation. The inverse relationship between Mg and Ca accumulation in leaves was found in GNT10 (Fig. 3).

The highest potassium content in the shoots was measured in GNT10 in variant B/100 (Fig. 3). Moreover, K accumulation in the shoots of GNT10 was reduced in the drought treatment, although it was still higher than in the fully irrigated control C/100. In the shoots of M × g and TV1, there were no significant changes in K concentration between the experimental variants. It is worth noting that all hybrids were characterised by a higher K concentration in the roots on metalliferous soil (Figure S1).

TME contamination reduced P accumulation in the shoots of all tested hybrids, and at the same time drought enhanced this effect (Fig. 3). A similar effect was observed for N concentration in the shoots (Fig. 3), although the combination of TMEs with drought caused a significant increase in nitrogen accumulation in the roots of all hybrids compared to the control at the same time (Figure S1).

### Principal component analysis and correlations

**Figure 4:**
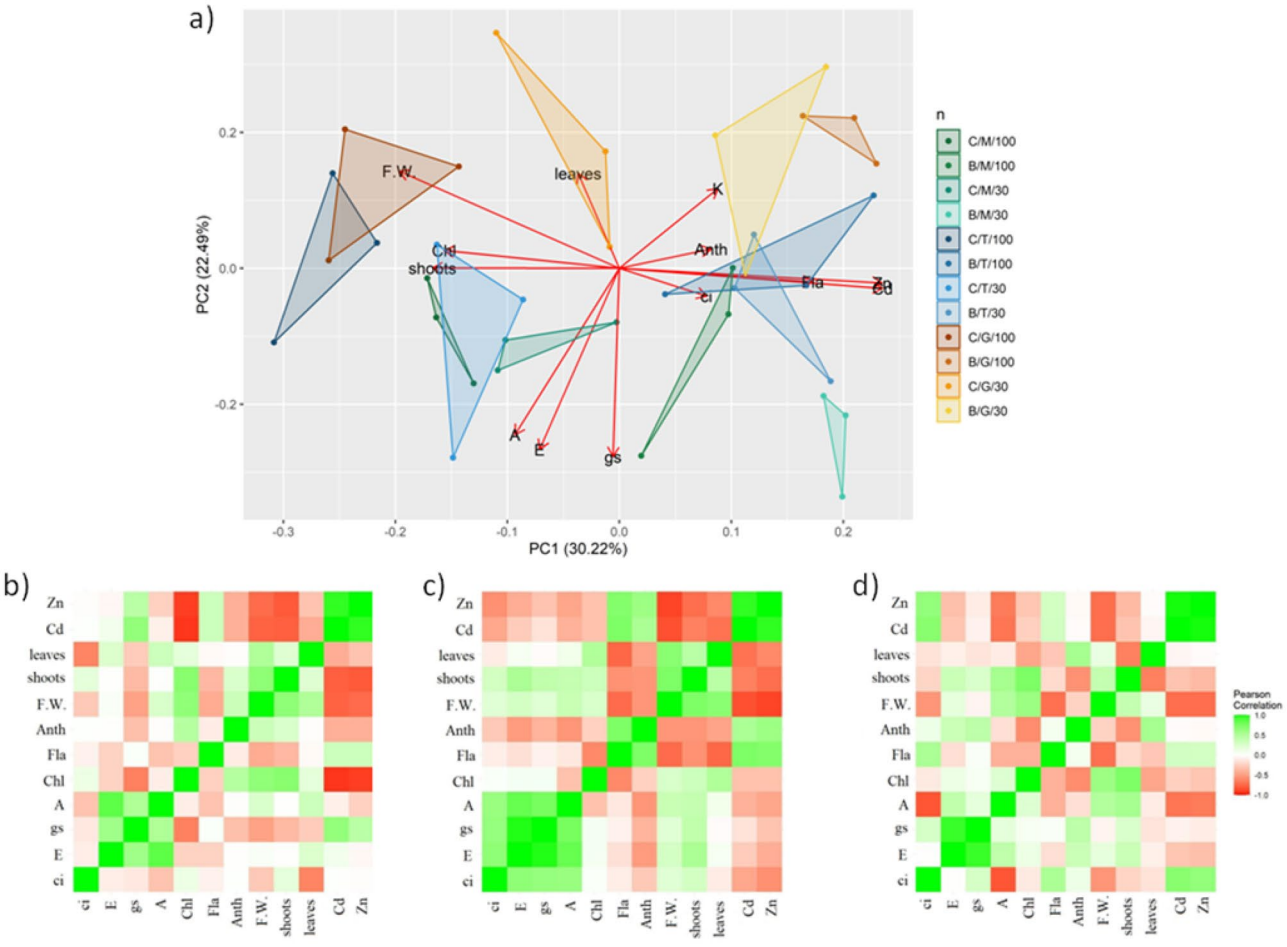
The principal component analysis (**a**) and heatmaps of correlation between selected parameters for M × g (**b**), TV1 (**c**), and GNT10 (**d**). Abbreviations for correlation heatmap: Ci—intercellular CO_2_ concentration; E—transpiration rate; gs—stomatal conductance; A—photosynthetic rate; Chl—chlorophyll content; Fla—flavonol content; Anth—anthocyanin content; F.W.—shoots fresh weight; n shoots—number of shoots per plant; n leaves—number of leaves per shoot; Cd—cadmium concentration in shoots; Zn—zinc concentration in shoots.

Graphical statistical analyses made it possible to characterise the responses of the hybrids studied to drought stress, metals, and their combination, and to indicate the variables that distinguish these responses (Fig. 4). GNT10 was characterised by the weakest gas exchange among the hybrids, which was compensated by the highest number of leaves and biomass, making it the most resistant to the effects of combined drought and TME contamination (Fig. 4A). The strongest correlations between the studied parameters were found for TV1, which might indicate a high sensitivity to TME contamination (Fig. 4C). For M × g and GNT10, the most important mechanisms for coping with stress seem to be biomass management (number of shoots and leaves) and gas exchange (Fig. 4B and D).

## Discussion

Drought resilience is an increasingly important and desirable trait, especially due to the devastating effects of climate change^22^. The response of plants to drought is well described in a wide range of species^23,24^. Species and hybrids cope with this stress in different ways. The regulation of leaf water potential by stomata can be described as a continuum between isohydric and anisohydric plant behaviour^19,25^. The anisohydric limit of this continuum could be described as a plant that maintains stomatal conductance despite drought and is thus insensitive to a decrease in soil water potential, while the opposite isohydric behaviour shows a rapid stomatal response when soil water potential decreases^26^. The classification into the isohydric to anisohydric continuum of the plant is usually done by water potential measurements and parameters derived from them^27,28^. However, there are other characteristics that allow locating the plant within the continuum, such as anatomical features, stomata morphology and density, and gas exchange measured in vivo^19,29,30^. The above groups of parameters are useful to assess the drought resistance of plants. For this purpose, the selection of plants that show a more isohydric behaviour in terms of stomata regulation is highly desirable for drought-prone marginal lands^25^.

The response of various *Miscanthus* hybrids to drought stress has been widely described^19,31,32^. However, from these reports, it could be concluded that M × g is generally more susceptible to drought stress compared to *Miscanthus sinensis*. It is worth noting that this might be related to the canopy architecture (lot of shots, low height) and the "stay green" phenotype. This observation has already been described by Rusinowski et al.^19^, who also confirmed a stronger anisohydric behaviour in *M*. × *giganteus* than in other *Miscanthus* hybrids tested in the field. Similar results were obtained in this work. Furthermore, the mechanism of interaction with drought stress varied considerably between the hybrids studied (M × g, TV1 and GNT10).

In this work, the effect of heavy metal stress (B) for each treatment was clearly visible in shoot biomass, physiological parameters and chlorophyll content. The effects of heavy metals on plants have been described in detail for hyperaccumulator model plants^33,34^ and for energy crop species, including the genus *Miscanthus*^6,10,21^. It is well known that energy grasses, despite their heavy metal exclusion properties^21^, can have significant negative effects on plant physiology and growth, especially in the first year of cultivation or in experiments under fully and semi-controlled conditions^35,36^. Mature plantations on soils contaminated with heavy metals can, depending on the nutritional status, resist the metals in the soil and produce a reasonable yield^21^.

Despite the growing interest in the effects of combined stress on plant growth and physiology, and in the mechanisms behind resistance to such combined stress, there is still a dearth of work showing the effects of combined stress from drought and heavy metals, and there is almost no work describing this phenomenon for energy crop species including *Miscanthus* hybrids^19^. This is an emerging topic, especially due to the fact that energy crops can only be grown on marginal land where food production is prohibited or economically prohibitive^37^. In this work, the combined effect of stress was most pronounced for each plant studied in the growth parameters. Ma et al.^38^ reported for *Brassica oxyrrhina* that heavy metals and drought individually and in combination caused a significant decrease in dry weight and an increase in lipid peroxidation products (MDA, malondialdehyde). However, for dry weight, the effect of metal and combined stress was lowest and at the same level, while MDA content was the same in each treatment. Similar observations regarding oxidative stress were also made in this work. Anthocyanin content in leaves, which has been shown to be strongly correlated with MDA content^39^, showed the same pattern for TV1, while GNT10 showed significantly higher levels only under combined stress and M × g induced anthocyanin production only under drought stress (C/30 and B/30).

From the results of the principal component analysis and the correlation matrices created separately for each hybrid (Fig. 4), it is clear that plants exposed to drought, heavy metals and a combination of these two types of stress show different responses. These mechanisms give a direct indication of which of the plants studied have a set of traits that enable them to grow and develop effectively in a harsh environment. Regardless of treatment, the most damaging factor to the plants was the presence of heavy metals in the soil. The observed reduction in shoot biomass yield was > 40% compared to the corresponding control, while the drought stress applied separately did not exceed a 30% reduction. The measurements related to gas exchange after drought application can largely explain the results of the shoot yields, considering the behaviour of the stomata in different plants.

The M × g showed a strong correlation between photosynthesis rate (A), stomatal conductance (gs) and transpiration (E) and the same parameters, which had almost the same values regardless of the treatment applied. This phenomenon could be explained by an anisohydric behaviour that keeps the stomata open and gas exchange at a high level until dehydration^40^. This statement was further confirmed by the highest decrease in shoot yield under drought and combined treatments. The significant effect of metals on this plant was visible in measurements of plant growth and chlorophyll content. Due to the applied stomata regulation strategy and morphological traits, M × g performed worst among the studied plants; however, a comparison with other studied plants may not be meaningful due to the specific experimental conditions (controlled environment, 1st year of cultivation).

*Miscanthus* hybrid TV1 seems to be more resistant to drought compared to M × g, which is reflected in the yield of the shoots. However, the damage observed from heavy metal stress in the soil was much more evident, and this was also true for the combined treatment. Interestingly, the correlation matrix for this species shows a correlation between the gas exchange parameters (A, gs, E), as in M × g, and an additional parameter correlated with the intracellular CO_2_ concentration (Ci). Nevertheless, TV1 showed a very strong correlation between anthocyanins, flavonols and heavy metals and a strong negative correlation between plant growth parameters, gas exchange parameters and heavy metals, showing the typical behaviour of plants under different types of abiotic stress^41^. From the point of view of stomata regulation, TV1 seems to occupy rather an intermediate position in the anisohydric-isohydric continuum of stomata regulation, but interestingly, this is only true for metal stress. De Silva et al.^42^ found in pot-cultivated red maple that heavy metal stress causes similar injuries in the xylem structure as drought, among other metal-specific injuries to the plant structure.

GNT10 showed a similar morphological response to trace metal element stress compared to TV1. However, this plant was the most resistant to the combined stress as shoot biomass did not change significantly between the separate stress application and the combined stress. This plant also shows a clear pattern of change when looking at the measurements of physiological parameters and element concentration, which is clearly visible in the correlation matrix. GNT10 showed a lower negative correlation between the measured parameters and the concentration of trace elements (Cd, Zn), which supports the hypothesis of a higher resistance to combined stress. When measuring gas exchange, it showed a clear pattern compared to the other plants. It was the only plant that showed a strong negative correlation between A and ci. This negative correlation was mainly associated with the strong effect of TME on the photosynthetic apparatus. Yang et al.^43^ observed a similar phenomenon in Cd-treated *Davidia involucrata*, suggesting that stomatal limitation is not the main factor affecting photosynthesis.

The results obtained on the mechanisms of heavy metal accumulation in different *Miscanthus* hybrids confirm the field observations reported by Rusinowski et al.^19^, but not directly. Rusinowski et al.^19^ showed that new *Miscanthus* hybrids with a more isohydric behaviour accumulated less trace metal elements, especially when droughts occur during cultivation. However, these data were collected on mature plantations and all plants studied produced the same amount of biomass. Pogrzeba et al.^44^ reported that *Miscanthus* plants accumulate much more elements in the first year of cultivation than in the following year. This could indicate a so- called dilution effect of trace elements in *Miscanthus* plants, which means that we detect less elements in higher amounts of biomass than in plants with lower amounts of biomass at the end of the season. Looking at GNT10, which showed the lowest effects of combined stress, followed exactly the pattern reported by Rusinowski et al.^19^: under drought conditions, lower amounts of metals were found in the aboveground biomass under drought conditions. However, this pattern was reversed in plants that were less resilient and where there was a huge decrease in biomass. However, when the total amount of metals in the biomass is calculated in all scenarios, the hypothesis made by Rusinowski et al.^19^ is confirmed for these specific plants.

## Methods

### Site description and soil sampling

The soil for the pot experiment was taken from two arable fields in Poland. The contaminated soil (lead, cadmium and zinc) came from farmland in Bytom (50° 20′ 41.9″ N 18° 57′ 19.9″ E), which is a short distance (about 2.5 km) from a former lead and zinc smelter. This smelter was in operation from 1927 to 1990 when significant soil contamination from dust fall occurred. Non-contaminated control soil was sampled near Pińczów (50° 32′ 35.3″ N 20° 29′ 4.0″ E), where cereals, legumes and root crops were grown in rotation in recent years. The soil collected at each site was air-dried and passed through a 4-mm sieve to remove stones and plant debris.

### Plant growth experiment

For each of the experimental treatments, four pots with a volume of 0.007 m^3^ were filled with 6.5 kg of air-dried control (C, for Pińczów) or contaminated (B, for Bytom) soil. Three *Miscanthus* hybrids were used for the experimental design—standard *Miscanthus* × *giganteus* (M × g), TV1 and GNT10. TV1 is an improved clone of the M × g type clone, and GNT10 is clonal interspecific hybrid^19^. M × g was acquired from Energene sp. z.o.o and is not subject to plant protection. TV1 was provided by Terravesta Poland. GNT10 was bred by Aberystwyth University/Ceres Inc in a programme compliant with the UN Convention on Biological Diversity (CBD), protected under CPVO title number 61030, licenced to Terravesta Ltd., with rhizomes provided by Terravesta Poland. The plant material was transferred on the basis of a material transfer agreement (MTA). All rhizomes came from three-year-old plantations on uncontaminated land. Individual rhizomes, weighing about 30–40 g, were planted in each pot. Plants were grown in a phytotron under controlled conditions: temperature 22/16 °C (day/night), light intensity PAR = 300 μmol (photons) m^−2^ s^−1^, photoperiod 16/8 h and humidity 50%. The experiment was conducted for five months at the Institute for Ecology of Industrial Areas. A total of 48 pots were planted with four replicates for each treatment. The treatments of the experimental design are listed in Table 3.

**Table 3:** Experimental treatmenxts.

UID	Soil	Hybrid	Water treatment (RWC) (%)
C/M/100	Non-contaminated	M × g	100
C/M/30	Non-contaminated	M × g	30
C/T/100	Non-contaminated	TV1	100
C/T/30	Non-contaminated	TV1	30
C/G/100	Non-contaminated	GNT10	100
C/G/30	Non-contaminated	GNT10	30
B/M/100	Contaminated	M × g	100
B/M/30	Contaminated	M × g	30
B/T/100	Contaminated	TV1	100
B/T/30	Contaminated	TV1	30
B/G/100	Contaminated	GNT10	100
B/G/30	Contaminated	GNT10	30

RWC, Relative Water Contet; C, control soil; B, contaminated soil; M, M × g; T, TV1; G, GNT10.

During the first 10-week growth period, a relative water content (RWC) of 100% was applied in all pots and monitored daily with the sensor WET (Delta-T Devices, UK). In the next phase of the experiment, irrigation was stopped for half of the pots in each treatment and after two weeks a RWC of 30% was reached. After a 4-week drought period, the photosynthetic rate, intercellular CO_2_, transpiration, stomatal conductance and pigment content of the plants were then measured in all pots. In the last four weeks of the experiment, the RWC was increased again to 100% in all pots. The number of shoots, height, diameter and number of leaves were then measured in each plant. The biomass was then harvested and divided into shoots, rhizomes and roots, washed in tap water and distilled water, dried and prepared for the element analyses.

### Soil physicochemical parameters and soil and plants elemental composition

All physico-chemical parameters of the soil were measured on the soil sieved through a 2 mm sieve. The pH of the soil was measured in H_2_O (ratio 1:2.5 m/v) and KCl using a combined glass/calomel electrode (OSH 10–10, METRON, Poland) and a pH-meter (CPC-551, Elmetron, Poland) at 20 °C. Electrical conductivity was determined with a ESP 2ZM electrode (EUROSENSOR, Poland) according to the Polish standard^45^. Soil texture was assessed using the hydrometric method according to the Polish standard^46^. Soil organic matter content (OM) was measured by loss on ignition as follows: 5 g of air-dry soil was dried at 105 °C for 24 h and then treated at 550 °C for 4 h.

Total concentrations of metals in soil (sieved through a 0.25 mm sieve) and in plants were analysed using a flame atomic absorption spectrometer (iCE 3500 FAAS, Thermo Scientific) after microwave sample digestion (ETHOS 1, Milestone, Italy) according to the procedure specified by the manufacturer (concentrated HNO_3_ and H_2_O_2_, 4:1 v/v). The total nitrogen concentration in the soil was determined by the dry combustion method^47^. The concentrations of available phosphorus and available potassium were determined according to the method described by Egnér et al.^48^. Total nitrogen (N) concentration in plants was measured by the titration method^49^ (Bremner, 1996), while total phosphorus (P), potassium (K), calcium (Ca) and magnesium (Mg) concentrations in plants were determined in previously mineralised samples by ICP (Liberty 220, Varian, USA). The sulphur (S) content was determined according to the Polish method^50^.

Gas exchange measurements were performed with an infrared gas analyser (LCpro SD, ADC Bioscientific UK). Saturated photosynthetic rate (A), transpiration rate (E) and stomatal conductance (gs) were measured as previously described by Rusinowski et al.^39^. The LCpro SD was equipped with a narrow chamber (580 mm^2^) set at 22 °C, 1500 μmol m^−2^ s^−1^ for photosynthetically active radiation (PAR) and a CO_2_ concentration of approximately 400 ppm. The relative chlorophyll content and the anthocyanin and flavonol pigments (relative units) were measured with a plant pigment content metre (Dualex Scientific + , Force-A, France).

## Supplementary Information

Supplementary Figures.

## Data Availability

Data available on reasonable request from corresponding author.
