# Supplementary material for: The effect of combined drought and trace metal elements stress on the physiological response of three Miscanthus hybrids
Source: Sci Rep. 2023 Jun 28;13:10452. doi: 10.1038/s41598-023-37564-5 (PMC10307809; doi:10.1038/s41598-023-37564-5)
Supplement: Supplementary file 1 — Supplementary Figures. [file 41598_2023_37564_MOESM1_ESM.pdf]

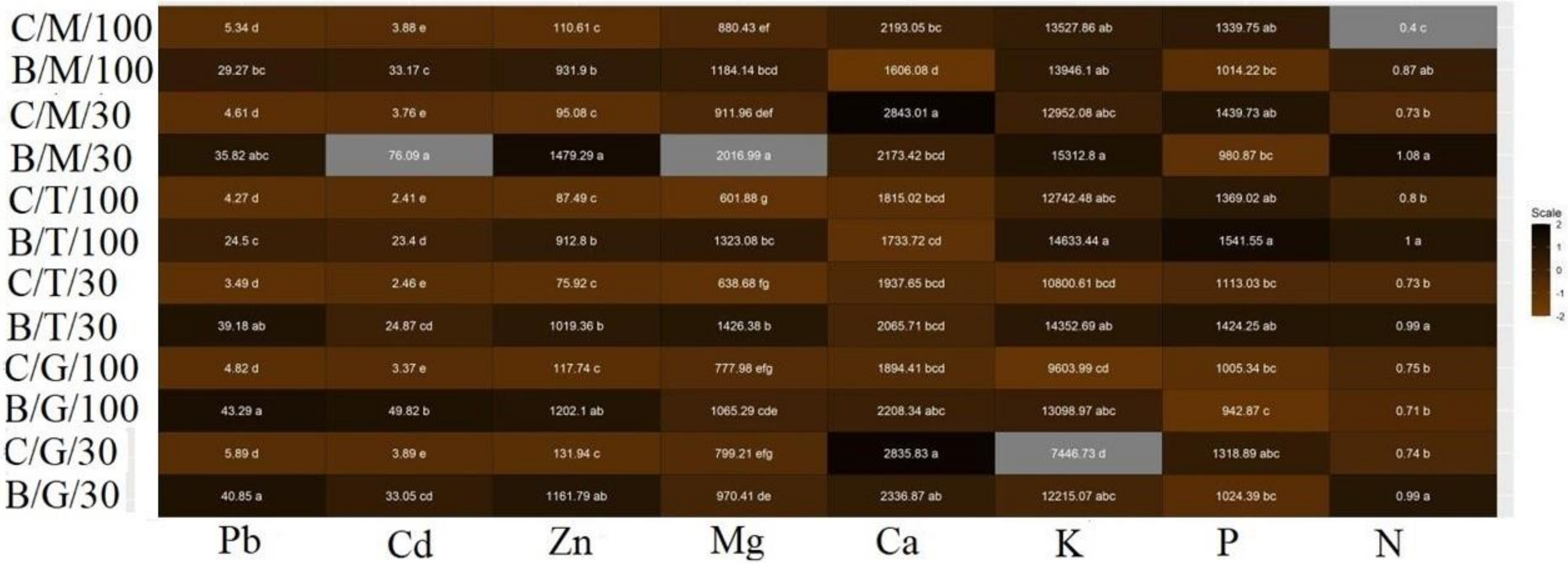

Figure S1. The effect of drought (/30) or/and TME contamination (B/), versus fully watered uncontaminated control soil (C/100) on accumulation of elements in roots (μg g^{-1} DW) of three tested *Miscanthus* hybrids. Presented values are means ± SE (n = 4).

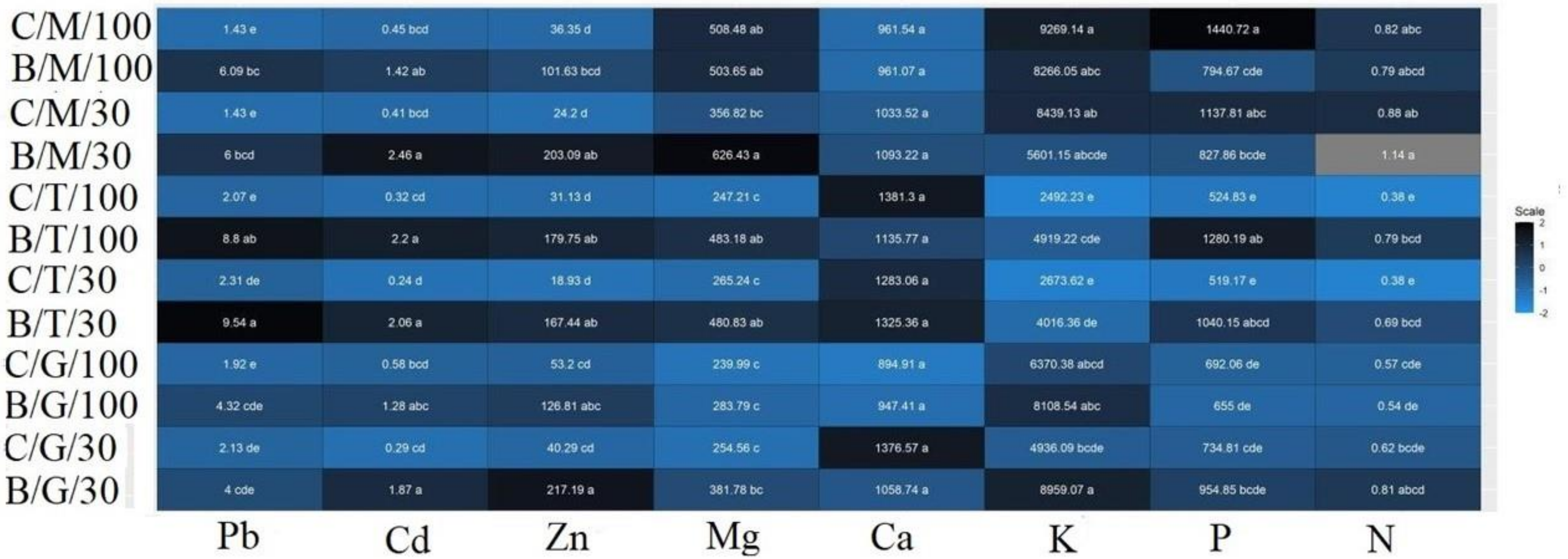

Figure S2. The effect of drought (/30) or/and TME contamination (B/), versus fully watered uncontaminated control soil (C/100) on accumulation of elements in rhizomes (μg g^{-1} DW) of three tested *Miscanthus* hybrids. Presented values are means ± SE (n = 4).
